# Group size planning for breedings of gene-modified mice and other organisms following Mendelian inheritance

**DOI:** 10.1038/s41684-023-01213-1

**Published:** 2023-07-24

**Authors:** Vladislava Milchevskaya, Philippe Bugnon, Emiel B. J. ten Buren, Dominique Vanhecke, Frank Brand, Achim Tresch, Thorsten Buch

**Affiliations:** 1 Cologne Excellence Cluster on Cellular Stress Responses in Aging-Associated Diseases (CECAD), University of Cologne, Cologne, Germany; 2 Institute of Medical Statistics and Computational Biology, Faculty of Medicine, University of Cologne, Cologne, Germany; 3 Institute of Laboratory Animal Science, University of Zurich, Zurich, Switzerland; 4 Quantitative Methods, Department of Business and Economics, Berlin School of Economics and Law, Berlin, Germany

**Keywords:** Animal breeding, Statistical methods, Ethics, Haplotypes, Policy

## Abstract

Colony management of gene-modified animals is time-consuming, costly and affected by random events related to Mendelian genetics, fertility and litter size. Careful planning is mandatory to ensure successful outcomes using the least number of animals, hence adhering to the 3R principles of animal welfare. Here we have developed an R package, accessible also through an interactive public website, that optimizes breeding design by providing information about the optimal number of breedings needed to obtain defined breeding outcomes, taking into account specific species, strain, or line properties and success probability. Our software also enables breeding planning for balanced male-to-female ratio or single-sex experiments. We show that, for single-sex designs, the necessary number of breedings is at least doubled compared to the use of all born animals. While the presented tool provides preset parameters for the laboratory mouse, it can be readily used for any other species.

## Abstract (web summary)

The authors developed an R package that enables the estimation of group size required for mouse breeding, taking into account Mendelian genetics, fertility and litter size.

## Main

Animal-based studies are essential for biomedical research. Accordingly, work with gene-modified rodents, mostly mice, has undergone an explosive development. Today a wide array of different mouse strains and genetically defined lines are used in biomedical research worldwide. Mouse Genome Informatics counts 64,000 entries for mouse lines^1,2^. A total of 12.6 million rodents per year are used for the creation and maintenance of gene-modified lines in the European Union alone^3,4^. Now, most projects require mice with combinations of modified alleles and not only single mutants. Such models facilitate precision research by answering scientific questions regarding gene function in organs, cell types and their interaction with age and environment. Obtaining mice with complex genotypes requires targeted breeding strategies. Some of the animals born from such breedings cannot be used for research or further breeding because they do not carry a genotype that can be experimentally exploited. Such animals are usually killed since gene technology legislation prohibits any use of gene-modified animals outside of science. While it is not possible to altogether avoid such surplus animals^3^, efforts are being made to keep the number of animals required for a breeding program as small as possible for ethical, practical and financial reasons. In some legislations, such breeding optimization is even a legal requirement^4^.

The practice of killing animals because they do not carry specific traits or are not needed has come under scrutiny in laboratory animal science^3–8^, farming^9,10^ and zoos^11–14^. The causes of unwanted surplus animals in laboratory animal facilities have been identified and include genetics of breeding, sex preference and the inability to match supply with demand^6^. Unfortunately, the frequency of expected genotypes according to Mendelian genetics^15,16^ does not deterministically translate into actual breeding outcomes. Stochastic fluctuations in allele distribution, fertility (some breeding pairs will produce no offspring), in utero viability of the different genotypes, and litter size (number of pups born or weaned per litter) have a large influence on breeding outcomes. Neglecting these factors often results in unnecessary breeding delays and scientifically unjustified animal use. In this Article, we describe a software package that enables researchers to plan mouse breeding projects based on a given success probability, derived from Mendelian genetics, fertility and litter size, and that integrates these parameters together with their stochastic effects in a probabilistic framework.

## Results

### Components of breeding outcome prediction and their stochastic behavior

**Fig. 1:**
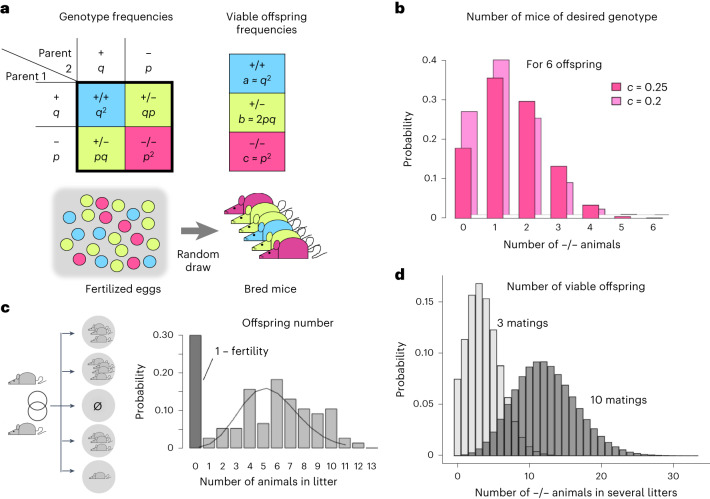
Stochastic effects in breeding. **a**, Top: Mendelian model of the outcome of a single breeding of two heterozygous animals. + and − denote different gene alleles occurring with probabilities *q* and *p*, respectively. Theoretical frequencies of the +/+, +/− and −/− offspring are *q*^*2*^, *2pq* and *p*^*2*^. Bottom: an outcome of single breeding is depicted as a random draw from the theoretical distribution above. **b**, Probability that out of six offspring exactly *X* animals will have the desired −/− genotype, given the probability *c =* *p*^*2*^ of the −/− genotype is 0.25 or 0.2. **c**, Example of the number of animals born out of a single breeding when taking into account fertility: there is a non-zero chance that 0 animals are born (1 − fertility). **d**, Distribution of −/− animals born for a genotype frequency of 0.25, for 10 (gray) or 3 (light gray) breedings, respectively. Source data

Typically, setting up breedings of mice harboring genes of interest located on different chromosomes is planned with the help of the Punnett square (Fig. 1a), which is based on Mendel’s laws of inheritance. It yields expected genotype frequencies of offspring from genetically defined parents^16^ (Fig. 1a), and various online Punnett square calculators are available to determine such allele frequencies^17,18^. It should be kept in mind that, if the breeding outcome does not follow classical Mendelian frequencies (for example, due to embryonal deaths^19,20^), the probabilities of occurrence may need to be adjusted from the Mendelian frequencies (Fig. 1b,c, 0.2). For instance, given a fixed litter size, the number of mice in any given litter that are homozygous for the null allele of a gene of interest (−/−) from parents that are heterozygous (+/−) for that gene follows a Binomial distribution with a Mendelian success probability of *c* = 0.25 (Fig. 1b). The actual number of successes (for example, −/− mice) observed in a single litter or small samples may thus differ substantially from the expected Mendelian outcome due to such unavoidable random fluctuations. Furthermore, litter size itself is a variable that can either be a positive number (size of the litter when the breeding is successful) or zero when the breeding is unsuccessful. The frequency of breeding success (productive breeding pairs) is known as fertility and depends on strain and husbandry conditions^21^ (Fig. 1c). To model the breeding process mathematically, we collected data from eight different mouse strains/lines, bred at the Laboratory Animal Service Center of the University of Zurich, to obtain the empirical distributions of the respective litter sizes. Considering only successful breedings, we found that the litter size distribution of most strains could be approximated by a Poisson distribution (Fig. 1c and Supplementary Fig. 1). The fraction of successful breedings (fertility) for each mouse strain, as included in our calculator, is obtained from the values reported by The Jackson Laboratory^21^. An additional parameter to be taken into consideration is the effective fertility, which comes into effect when the age of the experimental cohort is fixed to a short time interval, such as birth within 1, 2 or 3 days (ref. ^22^).

### Prediction of the required number of breeding pairs for single target genotypes

**Fig. 2:**
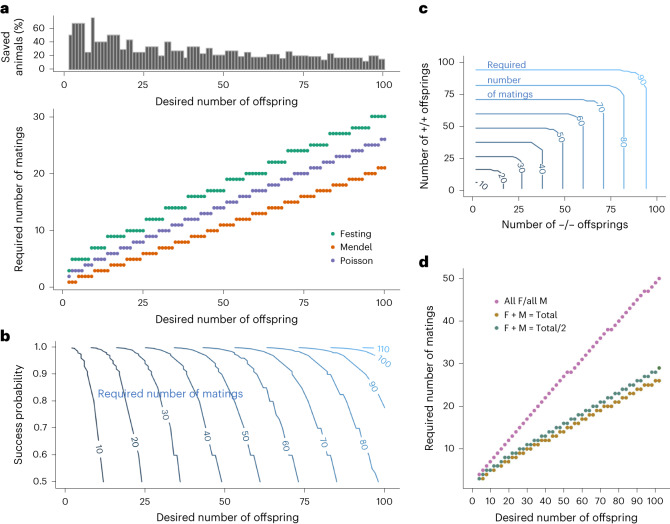
Performance of breeding models, in the case of an average litter size of 7 and a mouse fertility of 70%. **a**, Bottom: minimal number of breedings (*y* axis) needed to obtain the desired number of offspring (*x* axis) with 90% confidence, as calculated by three methods: the naive expectation due to Mendelian frequency (set to 100%; Supplementary Methods), the gold-standard textbook model suggested by Festing, and our method, denoted as Poisson. Top: relative surplus of breeding (in %) obtained by the textbook method (Festing), measured against our method (bottom). **b**, Minimal number of breedings needed to obtain a certain number of offspring (*x* axis) with a defined probability of success (*y* axis) using the Poisson method. **c**, Minimal number of breeding (contour lines) required in a setup where groups with two different genotypes need to be produced by the same breeding (90% confidence). The required numbers of animals from the two genotypes, that appear at a frequency of 25% each, are denoted on the *x* and *y* axis. **d**, Minimal number of breeding required to obtain offspring of specific sexes with 90% confidence, given that both female (F) and male (M) pups are born with equal probability. Shown are three scenarios: required *X* offspring need to be of the same sex (only *X* males or *X* females (purple)); *X* offspring can be of any sex (brown); required *X* offspring need to be balanced cohorts of each sex (that is, *X*/2 male and *X*/2 female pups, green). Source data

Having specified the components required for a group size prediction of a genetically defined animal breeding program, we next derived the distribution of the target offspring number as a function of the number of breedings (Fig. 1d and Supplementary Fig. 2). The probability of successfully obtaining the desired number of pups with the genotype of interest from a specific combination of parental animals can then be quantified. In the 1980s, M. Festing proposed a method for modeling the probabilistic outcomes in fertility and litter size^22^. Based on the distribution of the target offspring number, we can perform power and sample size calculations that prove to be more accurate than previous methods (Supplementary Methods). Further, our solution reduces the number of required breedings for obtaining a specific breeding outcome compared to the solution described by Festing (Fig. 2a), as exemplified for a breeding program with 0.25 Mendelian outcome, a litter size of 7% and 70% strain fertility. These reductions can exceed 60%, indicating the magnitude of improvement that can be achieved using our method (Fig. 2a, top). We also show that the simplistic use of the expected target animal number derived from Mendel´s laws combined with average litter size underestimates the required number of breedings dramatically (Fig. 2a, Mendel), explaining the frequent reports of difficulties to obtain sufficient animals for a particular experimental setup (unpublished observation, T.B.) or the necessity to include an undefined ‘fudge factor’^23,24^. While breeding success close to 100% may seem to be optimal from a planning perspective, we do not recommend setting the desired success probability overly high (for example, above 0.95) since a further increase in confidence becomes increasingly costly in terms of additional breedings and hence animals. (Fig. 2b and Supplementary Fig. 3). For successful breeding pairs that are continued to be used, not the strain fertility but rather a manual fertility of 100% may be applied, thus leading to smaller numbers of required breedings.

### Prediction of the required number of breeding pairs for outcomes requiring multiple genotypes or sex

Often, multiple genotypes need to be produced by the same set of breeding pairs, for example, identical numbers of +/+ and −/− animals from +/− parents. To guarantee the same success probability as for the single target genotypes, additional breedings are required in such situations (Fig. 2c).

The same calculations apply to group-size planning for obtaining defined numbers of animals of both sexes. While some experimental designs require all animals to be of the same sex, alternative designs can include both sexes (and account for sex-specific effects)^25^. A group-size planning for the inclusion of both sexes at identical numbers increases the required breedings only slightly over the simple use of all males and females born, without a fixed ratio (Fig. 2d). However, when only one sex is required, the necessary number of breedings doubles^21^ (Fig. 2d) compared to the use of all born animals.

### The BreedingCalculator software package

To facilitate appropriately powered breeding for the user, we incorporated algorithms and data for sample size calculation into our R package ‘BreedingCalculator’, available at GitHub^26^.

**Fig. 3:**
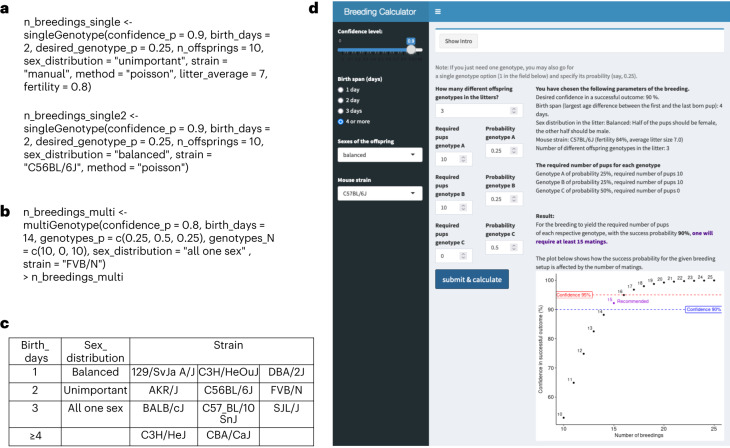
The breedingCalculator software package and the Breeding Calculator website. **a**, Shown is the use of the ‘singleGenotype’ function of the ‘breedingCalculator’ package in R. Example parameters are a power of 90%, a period of 2 days within which birth should occur, the number of required offspring, namely 10, a design accepting any male/female balance (top) and balanced sex design (bottom) with equal numbers of males and females required for the experiment, a frequency of the needed genotype of 25%, and manually added strain parameters (top; litter size of 7 and fertility of 80%) or use of strain information in the package (bottom, C57BL/6J). **b**, Shown is the use of the ‘multiGenotype’ function. Example parameters are a power of 80%, a period of 14 days within which birth should occur, a frequency of the genotypes in the litter, namely 25%, 25% and 50%, the number of required offspring from the two genotypes of interest (10 each) in a one-sex design and use of the FVB/N strain. **c**, The table shows important parameters that can be chosen in the ‘breedingCalculator’ package, such as birth span, sex distribution and strain. **d**, Screenshot showing the use of the Breeding Calculator website at https://www.ltk.uzh.ch/en/Breeding.html. Example parameters are a power of 90%, a period of >4 days within which birth should occur, a balanced sex design with equal numbers of males and females required for the experiment, the C57BL/6 strain, the number of different genotypes in the litter (3), the frequencies of the three genotypes (25%, 25% and 50%), and the number of pups required for the two genotypes needed (10 animals for each genotype). The result is indicated in purple. The graph indicates breeding success as the result of the chosen parameters as a function of the number of breedings. The 90% and 95% thresholds are indicated with the recommended 15 breeding pairs highlighted in purple.

When the experimental setup aims for offspring of a single genotype or simply at a total number of born pups, one may use the singleGenotype function to calculate the required number of breedings. The parameters ‘confidence, ‘birth_days’, ‘n_offsprings’, ‘sex_distribution’, ‘desired_genotype_p’, and ‘strain’ or ‘litter_average’ and ‘fertility’ may be defined (Fig. 3a,c and Box 1).

However, when the breeding setup aims to obtain offspring of multiple genotypes from the same breedings, it is better to use the multiGenotype function to calculate the required number of breedings. Here the distribution of the predicted frequencies of the genotypes is given through ‘genotypes_p’ and the additional parameter ‘genotypes_N’ allows the researcher to specify how many animals of each genotype are needed (Fig. 3b,c). The ‘method’ should be usually set to ‘poisson’ unless the aim is to compare the results with the published method from M. Festing (method set to ‘festing’). The number of pups expected to be born can be obtained by the function expectBorn. Finally, confidence changes, as a function of the number of breedings, can be visualized by probabilitiesPlots to facilitate finding optimal parameters. The breeding calculator supports a standardized output into pdf format for documentation. We provide simplified, interactive access to this package on the website https://www.ltk.uzh.ch/en/Breeding.html (Fig. 3d)^27^.

Box 1 Definition of parameters used in the breeding calculator. We refer here in general to the online calculator. The respective parameters of the R package are indicated in parenthesis and italics Number of required animals (genotypes_N): the number of animals at weaning age that can be obtained with the set confidence.

Confidence: likelihood of successfully obtaining the required number of animals.

Mendelian success probability (desired_genotype_p): percentage of offspring of the desired genotype determined, for example, by the Punnett square. Likewise, deviations from the classical Mendelian percentages can be used (for example, due to partial fetal death).

Fertility: percentage of breeding couples of a particular strain/line that give birth to at least one litter and bring the pups to weaning age; if animals at different age need to be used (for example, fetuses, newborns or aged), this number needs to be adjusted accordingly.

Litter size (litter_average): the mean litter size at weaning age for a particular strain or line; if animals at different age need to be used (for example, fetuses, newborns or aged), this number needs to be adjusted accordingly.

Sex (sex_distribution): The researchers can choose to use all animals of either one sex or both sexes. In the latter case, they can choose to use either equal numbers of males and females (a balanced design) or all male and female animals.

If the sex ratio of a particular breeding deviates from the 50% norm, ‘all animals’ should be used in the sex settings. Sex can then be treated as a gene with two alleles in a heterozygous (males) with homozygous (females) cross and respective parameters set (for example, 0.4 and 0.6 for the two possible outcomes).

Birth span (birth_days)*:* this parameter gives the age span that the offspring produced from the breedings are allowed to have for use in subsequent experiments or other purposes. Unless the researchers want to perform a study with a particularly narrow age range (1, 2 or 3 days) the value should be set to 4 or more (however, the upper limit is approximately 3 weeks since after that period a next generation of pups may be born).

## Discussion

Optimization of breeding protocols for reduction of animal use is an ethical obligation mandated within the commonly applied 3R (replace, reduce, refine) principle^28^. Yet, the very basic biology of mammalian genetics and associated stochastic breeding processes inevitably create surplus animals that cannot be further used in experiments or breeding. We have developed an R package that supplies the optimal solution, that is the least number of required breeding animals, depending on required breeding outcomes and strain characteristics. Our algorithm uniformly performs better than previously published tables and schemes (for example, refs. ^22–24^). We removed from the workflow any form of guesswork commonly done by scientists to adjust for self-experienced randomness. Also, by using appropriate group-size calculations for breedings, experiments are more likely to be conducted as planned, thereby improving reproducibility and research efficiency, and reducing financial costs. At first glance, it may seem that powered breeding planning increases the number of animals produced for an experiment. But this is not the case, because in cases in which the planning is not adequate and the desired number of animals is not reached a new breeding round will be required. In this case, there is a risk that animals from the first round remain unused because, for instance, they would be too old or of too different age compared to the new cohort.

When experiments are performed with cohorts obtained through multiple breeding rounds, batch effects can be a threat. If such batch effects are expected to be negligible or can be corrected for, so-called adaptive designs with multiple breeding rounds and interim evaluations are an option to save, on average, even more animals. Such designs are common, for example, in clinical trials where costs per sample are high^29^. They can be adapted to the animal breeding process. Our software serves as a building block for such adaptive designs, as it can determine the probability of success (of obtaining enough animals) in each breeding round, which in turn is used to calculate the expected number of matings required. Optimal planning enables the required number of animals to be achieved while minimizing the number of excess breeding attempts. Incorporation of our software or the underlying calculations into husbandry software may facilitate even better planning of breeding in the future.

With our package, we aimed to cater to the needs of the large community of researchers that use gene-modified mice. Thus, we incorporated into the software package preset parameters for commonly used mouse strains. However, the calculator can also be used for other species, when the respective parameters are available. The calculator could in principle also be applied for trio breedings (one cage with two dams and one male), as these types of breeding usually exhibit similar performance to duo breedings^30–33^. The main concern lies in the possibility of subfertile or infertile males leading to reduced fertility values in our calculator; this can be particularly problematic in trio breeding where an infertile male will impact two breeding dams, which would affect the resulting outcome substantially. Since there is usually no information to which extent females or males cause unsuccessful breedings, this may be corrected simply by slightly decreasing the strain fertility (1% or 2%).

While the unequal use of sexes in animal experimentation has been a topic of discussion^34–36^ and statistical solutions regarding experimental designs have been suggested^25,37^, we here provide evidence that restricting experiments to one sex unnecessarily leads to additional breedings and hence unused offspring beyond a simple doubling.

In conclusion, we have developed a statistical method supported by software to accurately predict the minimal number of breeders required to obtain experimental or breeder cohorts. The method is readily accessible to the public via GitHub and a web application. Our solution thus facilitates the reduction of surplus animals during breeding and hence supports adherence to the 3R principles also in breeding.

## Methods

Methods are available in Supplementary Information.

### Reporting summary

Further information on research design is available in the Nature Portfolio Reporting Summary linked to this article.

## Online content

Any methods, additional references, Nature Portfolio reporting summaries, source data, extended data, supplementary information, acknowledgements, peer review information; details of author contributions and competing interests; and statements of data and code availability are available at 10.1038/s41684-023-01213-1.

## Supplementary information

Supplementary Information Supplementary Figs. 1–3 and Methods 1–4.

Reporting Summary

## Data Availability

The primary data from the curve fitting calculations on the litter size distribution are available at BioStudies, S-BSST1034 (https://www.ebi.ac.uk/biostudies/studies/S-BSST1034). The R package can be accessed at https://github.com/VladaMilch/breedingCalculator or directly from an R installation (library ‘breedingCalculator’). Source data are provided with this paper.

## Source data

Source Data Fig. 1 Source data for histogram in Fig. 1.
Source Data Fig. 2 Source data for Fig. 2a–d.
